# Global MicroRNA Expression Profiling of Mouse Livers following Ischemia-Reperfusion Injury at Different Stages

**DOI:** 10.1371/journal.pone.0148677

**Published:** 2016-02-09

**Authors:** Weisheng Zheng, Hewei Men, Jing Li, Yu Xing, Bin Wu, Zhenglu Wang, Junjie Li, Dahong Teng, Yuan Shi, Jiang Li, Pu Jiang, Jinzhen Cai

**Affiliations:** 1 Department of Transplant Surgery, Tianjin First Central Hospital, Tianjin, China; 2 The School of Life Sciences and Technology, Tongji University, Shanghai, China; 3 Department of Gastroenterology, Tianjin First Central Hospital, Tianjin, China; 4 Department of Forensic Medicine, College of Basic Medicine, Chongqing Medical University, Chongqing, China; 5 Tianjin Medical University, Tianjin, China; National Institutes of Health, UNITED STATES

## Abstract

Hepatic ischemia-reperfusion injury is a dynamic process consisting of two stages: ischemia and reperfusion, and triggers a cascade of physiological and biochemical events. Given the important role of microRNAs in regulating gene expression, we analyzed gene expression changes in mouse livers at sham control, ischemia stage, and reperfusion stage. We generated global expression profiles of microRNA and mRNA genes in mouse livers subjected to ischemia-reperfusion injury at the three stages, respectively. Comparison analysis showed that reperfusion injury had a distinct expression profile whereas the ischemia sample and the sham control were clustered together. Consistently, there are 69 differentially expressed microRNAs between the reperfusion sample and the sham control whereas 28 differentially expressed microRNAs between the ischemia sample and the sham control. We further identified two modes of microRNA expression changes in ischemia-reperfusion injury. Functional analysis of both the differentially expressed microRNAs in the two modes and their target mRNAs revealed that ischemia injury impaired mitochondrial function, nutrient consumption, and metabolism process. In contrast, reperfusion injury led to severe tissue inflammation that is predominantly an innate-immune response in the ischemia-reperfusion process. Our staged analysis of gene expression profiles provides new insights into regulatory mechanisms of microRNAs in mouse hepatic IR injury.

## Introduction

There is severe shortage of donor livers per year [1]. The organ shortage has turned to the use of extended criteria donor livers including donor livers having been subjected to prolonged storage as well as from non-heart-beating donors. The common feature of these “marginal” donor livers is high susceptibility to ischemia-reperfusion injury. The ischemia-reperfusion injury may increase the early organ failure and the incidence of rejection after transplantation [2]. As a consequence, the survival rate of these “marginal” livers after transplantation is lower than the normal criteria donor livers. Therefore, fully understanding the molecular mechanism of hepatic ischemia-reperfusion injury would promote the use of these “marginal” donor livers in clinical surgeries.

A cascade of physiological and biochemical changes take place in hepatic ischemia-reperfusion injury [3]. In the ischemia stage, the oxygen and nutrient deprivation and metabolic disruption induce the mitochondrial dysfunction, and trigger the deficiency of energy production, which lead to the injury and death of liver parenchymal cell. In the reperfusion stage, the blood flows into the liver and exacerbates the liver injury by triggering a series of immune cells filtration, innate immune and inflammatory molecules activation, like Kupffer cells, Dendritic cells, Natural killer cells, TLR4, reactive oxygen species (ROS) and other cytokines [4, 5].

Previous studies identified a bunch of differentially expressed genes that mediated the physiological and biochemical events triggered by hepatic ischemia-reperfusion injury [6–8]. For example, Toll-like receptor 4 (TLR4) was overexpressed in liver transplantation. Down-regulation of TRL4 attenuated liver ischemia-reperfusion injury [9]. MicroRNAs are a class of short noncoding RNA molecules (21–30 nucleotide long) widely endogenously expressed in plants, animals, and viruses [10–12], and mainly function posttranscriptionally through mRNA decay and translational repression by base-pairing to the 3’ untranslated regions of target mRNAs [10, 13–15]. Recent studies have uncovered a regulatory role of microRNAs in ischemia-reperfusion injury in organ transplantation surgery. For example, 40 differentially expressed microRNAs associated with proinflammatory et al. processes were identified in ischemia-reperfusion injury post-liver transplantation [16]. Nine microRNAs were differentially expressed in renal ischemia-reperfusion injury [17]. miR-223 was up-regulated in the hepatic ischemia-reperfusion injury [18]. In contrast, miR-146a was down-regulated in the early stage of hepatic ischemia-reperfusion injury [19]. Seventy-eight microRNAs with more than two fold expression difference were identified in the mice livers upon ischemia-reperfusion injury [20].

Previously identified microRNAs associated with hepatic ischemia-reperfusion injury mainly focused on individual ones. There is no global study to screen for the microRNAs in response to hepatic ischemia-reperfusion injury. Needless to say, there are no studies on altered regulation of microRNAs in the ischemia stage and the reperfusion stage, respectively. Therefore, how hepatic microRNAs respond to ischemia-reperfusion injury largely remains elusive. In this study, we profiled expression of both microRNAs and mRNAs in the mouse livers subjected to sham operation, warm ischemia (WI), and ischemia followed by reperfusion (IR), respectively. We further performed clustering analysis of the expression profiles, identified differentially expressed (DE) genes pairwisely, and interrogated their functions and the mechanisms by which microRNAs respond to hepatic ischemia-reperfusion injury through regulating their target genes. Our results show that IR injury leads to a relative distinct expression profile whereas expression profiles of the sham sample and the WI sample are clustered together. MicroRNAs respond differently to WI and IR injury by different sets of DE microRNAs with different functions. Particularly, miR-125b-5p and miR-501-3p are down-regulated and activate the Toll-like receptor signaling pathway in response to hepatic IR injury.

## Materials and Methods

### Animals

Male C57BL/6J mice (8–10 weeks old, weighing 15–18 g) were obtained from PLA Military Academy of Medical Sciences Laboratory Animal Center. Animals were housed in a stainless steel with *ad libitum* access to filtered tap water and commercial feed in a specific pathogen free animal room in the supervision and testing center with 12 h light-dark cycles. Mice were fasted for 12h before the experiment but allowed free access to water. All experimental procedures involving animals were carried out in accordance with the National Institutes of Health Guide for the Care and Use of Laboratory Animals after review and approval by the Ethics Committee of Tianjin Medical University.

### Hepatic ischemia/reperfusion (I/R) model

The mice were randomly divided into three groups of six mice each: (i) the sham group underwent a sham operation without vascular occultation; (ii) the warm ischemia (WI) group with hepatic vascular occultation for 90 min; and (iii) the ischemia followed by reperfusion (IR) group underwent vascular occultation for 90 min followed by 120 min of hepatic reperfusion. No mice died before the following surgery.

Specifically, the established non-lethal model of segmental (70%) hepatic warm ischemia/reperfusion was performed as previously described [21]. Surgery was performed under anesthesia by an intraperitoneal injection of 4% of chloral hydrate (0.3 ml/100g, Sigma-Aldrich, P3761, St. Louis, MO, USA). During anesthesia, body temperature was maintained between 36.5 and 37.5°C by a heating pad and lamp by monitoring with a rectal probe. A midline laparotomy was done for all mice. For the WI and the IR group, all structures in the portal triad (hepatic artery, portal vein, bile duct) to the left and median liver lobes were occluded with a microvascular clamp (Fine Science Tools) for 90 min. The liver samples of the WI group mice were obtained for further analysis (histopathology, microRNA- and RNA-seq). For the IR group, reperfusion was initiated by removal of the clamp. The abdominal incision was then closed with 3–0 polypropelene sutures. During reperfusion, the mice were kept in clean cages with no further administration of anesthetic. After 120 min of reperfusion, the liver samples of the IR group mice were obtained for further analysis. For the Sham group, after a midline laparotomy, kept in clean cages for 90 min, the same amount of time for ischemia operation, the liver samples of the sham group mice were obtained for further analysis. All mice were sacrificed with administration of anesthetic after I/R operations.

### Histopathology

Formalin-fixed liver samples were embedded in paraffin and cut to 6-μm-thick sections. Liver tissues were stained with hematoxylin-eosin, and slides were assessed for inflammation and tissue damage.

### RNA isolation

The obtained liver tissues were minced quickly into very small pieces and frozen in liquid nitrogen. RNA was extracted from 130–150 mg of liver tissue for each group mice using TRIzol RNA isolation kit (Ambion, Invitrogen, Carlsbad, CA, USA) following the manufacturer’s instruction. The quality and integrity of the purified RNA was assessed using a BioAnalyzer 2100. Only total RNA samples with RIN>7.5, concentration>200 ng/μl were retained for sequencing library construction.

### Quantitative PCR analysis

Reverse transcription was performed using a reverse-transcription system (Promega) according to the manufacturer’s protocols. Quantitative RT-PCR was performed using KAPA SYBR Fast qPCR kit (TAKARA, Code: DRR036A) and signals were detected with ABI 7500 Real-Time PCR System (Applied BioSystems). GAPDH was used as endogenous control. The RT-PCR was carried out in triplicate. All the primers used are listed in S1 Table.

Realtime quantification of microRNAs was carried out by stem-loop RT-PCR as described before [22]. Briefly, reverse transcription was performed using a reverse-transcription kit (TAKARA, code: DRRO47A) according to the manufacturer’s protocols. Quantitative RT-PCR was performed using KAPA SYBR Fast qPCR kit (TAKARA, Code: DRR420A) and signals were detected with ABI 7500 Real-Time PCR System (Applied BioSystems). RnU6 was used as endogenous control. The RT-PCR was carried out in triplicate. All the primers used are listed in S2 Table.

### RNA- and microRNA-sequencing

RNA-seq sequencing libraries were constructed from the extracted RNA using standard Illumina libraries prep protocols. RNA-seq was performed on Illumina HiSeq2000 platform.

For microRNA-sequencing, we extracted small RNAs by size-fractionated total RNA on a denaturing polyacrylamide gel. We then ligated the proprietary 3’- and 5’-adapters to the small RNAs using truncated T4 RNA ligase. The adapter-ligated RNAs were used as template to synthesize cDNA using Superscript III reverse transcriptase. The cDNA was amplified by PCR to generate cDNA libraries. Then, the libraries were sequenced on Illumina HiSeq2000 platform.

Sequencing reads have been deposited in the National Center for Biotechnology Information’s Gene Expression Omnibus (GEO) under the accession number GSE72315.

### RNA-seq and microRNA-seq data analysis

For small RNA sequencing reads, we first cleaned the raw sequencing reads by removing adaptor sequences, low quality reads and size selection (15~30 nt). Next, the clean reads were mapped to the mouse mm9 genome by using Bowtie v.0.12.7 with parameters: -m 5 -n 1 -e 80 -l 30 -a -m 5—best—strata. Then the alignments were classified into different types of RNA (rRNA, snRNA, snoRNA, microRNA, etc.) using HTSeq with the parameters: -m intersection-nonempty -q -t exon -s no and mouse RNA annotations were downloaded from Rfam, Ensembl, Gencode, and miRBase. We retained the clean reads mapped to microRNAs, and used miRDeep2 pipeline[23] to get the mature miRNA expression profiles. Then the numbers of microRNA reads were normalized as Trimmed Mean of Mvalues (TMM) = (read counts assigned to a microRNA) / (scaling factor x total read counts) x 10^6^ using Bioconductor package edgeR. Scaling factor was generated by edgeR for each sample. Total read counts equals to the number of mapped reads in a sample. Differentially expressed microRNAs were calculated by the criteria of fold-change > 1.5 and Fisher exact test p-value < 0.01 in pairwise comparisons.

For mRNA sequencing reads, we mapped reads to mouse mm10 genome and splicing junction annotations by using Tophat v2.0.9 with default parameters. Then the alignment results were retained to assemble into gene transcripts and calculate expression level of each gene. The gene expression level was normalized as Fragments Per Kilobase per Million mapped reads (FPKM). Differentially expressed mRNA genes were selected from pairwise comparisons by using Cuffdiff v2.0.2 with default parameters.

### Biological function analysis of identified gene sets

We used Ingenuity Pathway Analysis software (IPA, http://www.ingenuity.com) to investigate the functions and involving pathways of differentially expressed genes (microRNAs and mRNAs) in the mouse hepatic ischemia-reperfusion injury. We further built molecular interaction networks of the genes to reveal their regulatory mechanisms.

## Results

### Hepatic injury caused by ischemia and reperfusion

**Fig 1:**
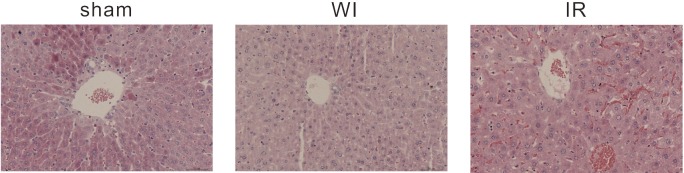
Representative hematoxylin and eosin staining examination of liver sections. In the sham sample, hepatocyte cords were clearly arranged in periportal area. No histological abnormalities are observed. In the WI sample, there are slight edema and partial steatosis of hepatocyte in the central venous area. In the IR sample, there are significant congestion of sinusoid, obvious hepatocyte edema and steatosis. Hepatocyte cords were arranged disorderly. (Original magnification 200x).

Histological examination shows hepatic injury after treatment of warm ischemia (WI) and ischemia followed by reperfusion (IR) (Fig 1). We observed slight edema and partial steatosis of hepatocyte in the central venous area in the mice treated with WI. The hematoxylin and eosin staining examination in the mice subjected to IR injury manifested severe hepatocyte edema and steatosis, and significant congestion of sinusoid. Moreover, hepatocyte cords were arranged disorderly. In contrast, hepatocyte cords were clearly arranged in periportal area in the sham-operated mice. And we did not find histological abnormalities. The similar hepatic injury was observed in the mice or rats subjected to IR treatment in the previous studies[18–20]. These results suggest that our mouse model of hepatic WI and IR injury was successfully established.

### Disturbance in gene expression by hepatic IR injury

**Fig 2:**
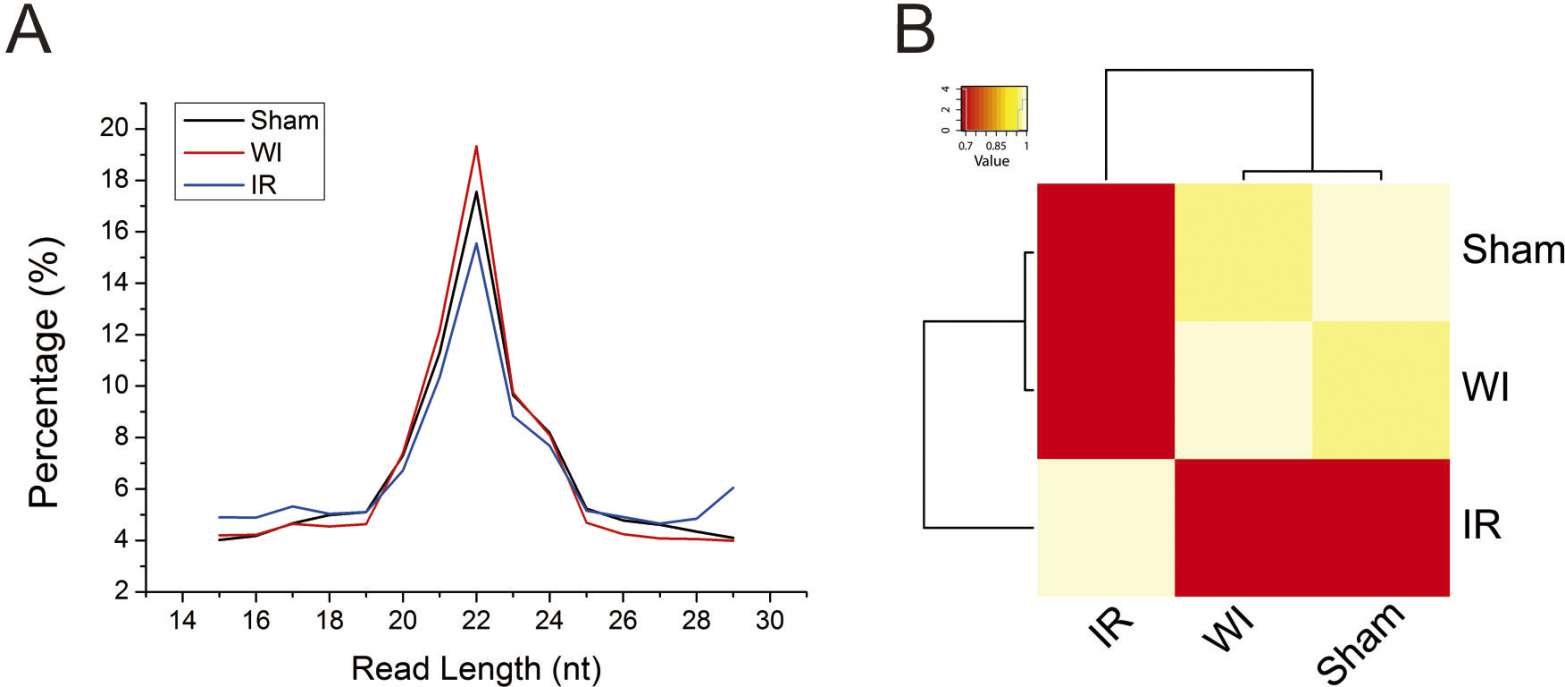
Expression profiles of microRNAs. (A) Length distribution of microRNA reads. (B) Unsupervised hierarchical clustering of expression profiles of microRNAs. Sham: control, WI: warm ischemia, IR: ischemia followed by reperfusion.

To gain insights into the molecular mechanisms of hepatic injury in mice subjected to IR treatment, we profiled the expression of mRNA and microRNA genes in the three samples using RNA-seq. The number of reads are summarized in the Tables 1 and 2. The length of microRNA reads has a range of 15–29 nt with a peak at 22 nt for all three samples, suggesting the homogeneity of the samples (Fig 2A). The unsupervised hierarchical clustering of the microRNA expression profiles show that the warm ischemia sample and the sham sample are clustered together (Fig 2B). This suggests that the WI sample has more similar expression profiles to the sham sample than to the IR sample. The difference in the expression profiles between the three samples is consistent with the histological examination results that the IR sample displayed more severe injury (Fig 1).

**Table 1:** Statistics of mRNA-seq reads.

Sample	Clean read pairs	Uniquely aligned concordant pairs
Sham	30006373	21508594 (71.7%)
WI	32668027	23602541 (72.2%)
IR	30717065	19460482 (63.3%)

**Table 2:** Statistics of small RNA-seq reads.

Sample	Clean reads	Reads aligned to genome
Sham	16291554	10760457 (66.05%)
WI	17700730	11864493 (67.03%)
IR	22618844	11676029 (51.62%)

**Fig 3:**
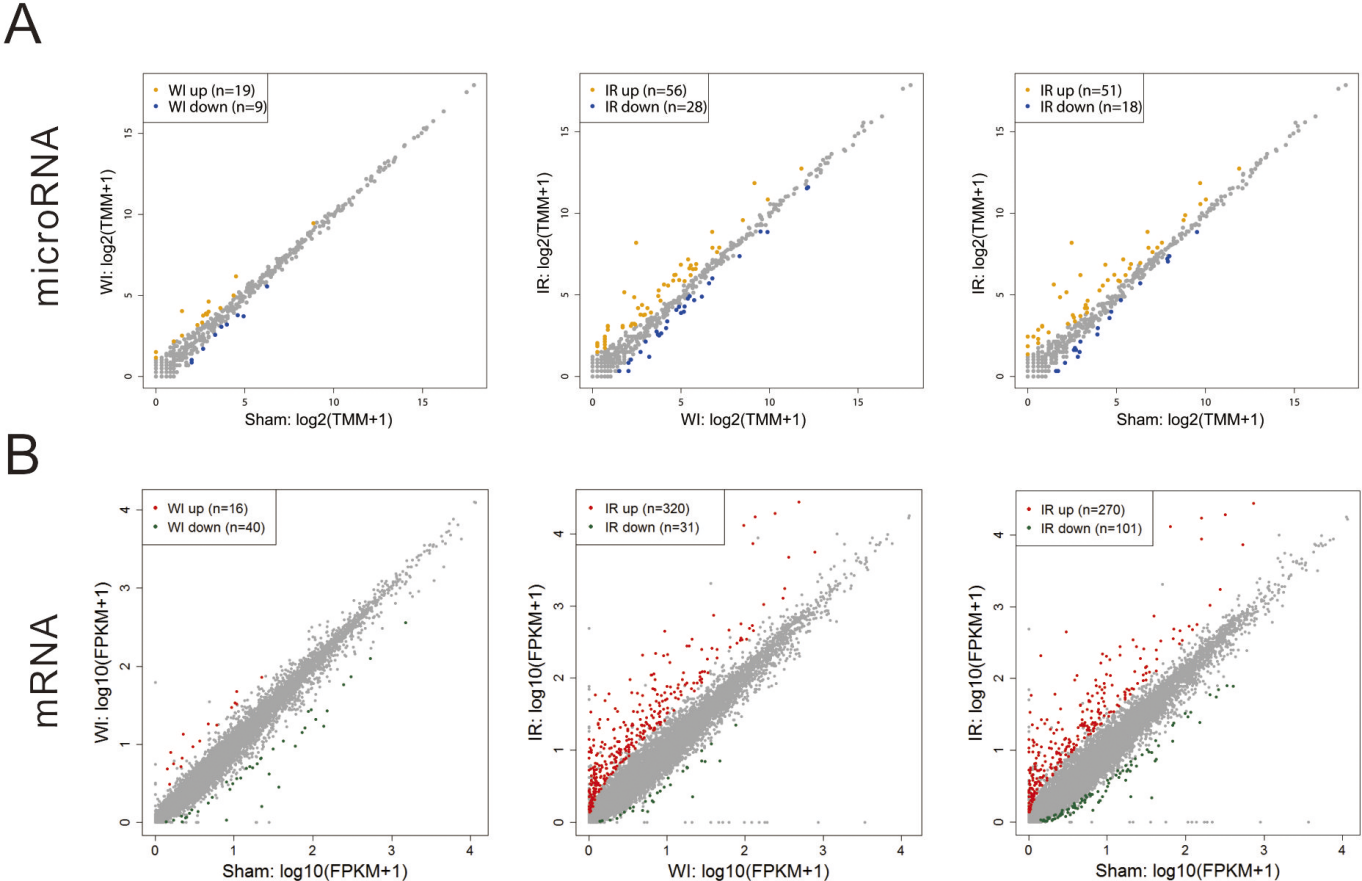
Differentially expressed genes. Scatter plots showing the pairwise comparisons of microRNA (A) and mRNA (B) expression profiles. Color dots indicates the differentially expressed genes.

We further identified the differentially expressed (DE) genes by pairwise comparisons. As expected, there are small number of DE genes between the WI sample and the sham sample whereas there are many DE genes when comparing the IR sample to the WI sample or the sham sample (Fig 3). The DE microRNA and mRNA genes are listed in S3 and S4 Tables, respectively. This further suggests that reperfusion injury resulted in a more extensive disturbance in gene expression when compared to ischemia injury. The realtime quantitative PCR results confirmed the expression changes of the DE genes (S1 Fig).

### Regulatory networks of DE microRNAs and mRNA genes

**Fig 4:**
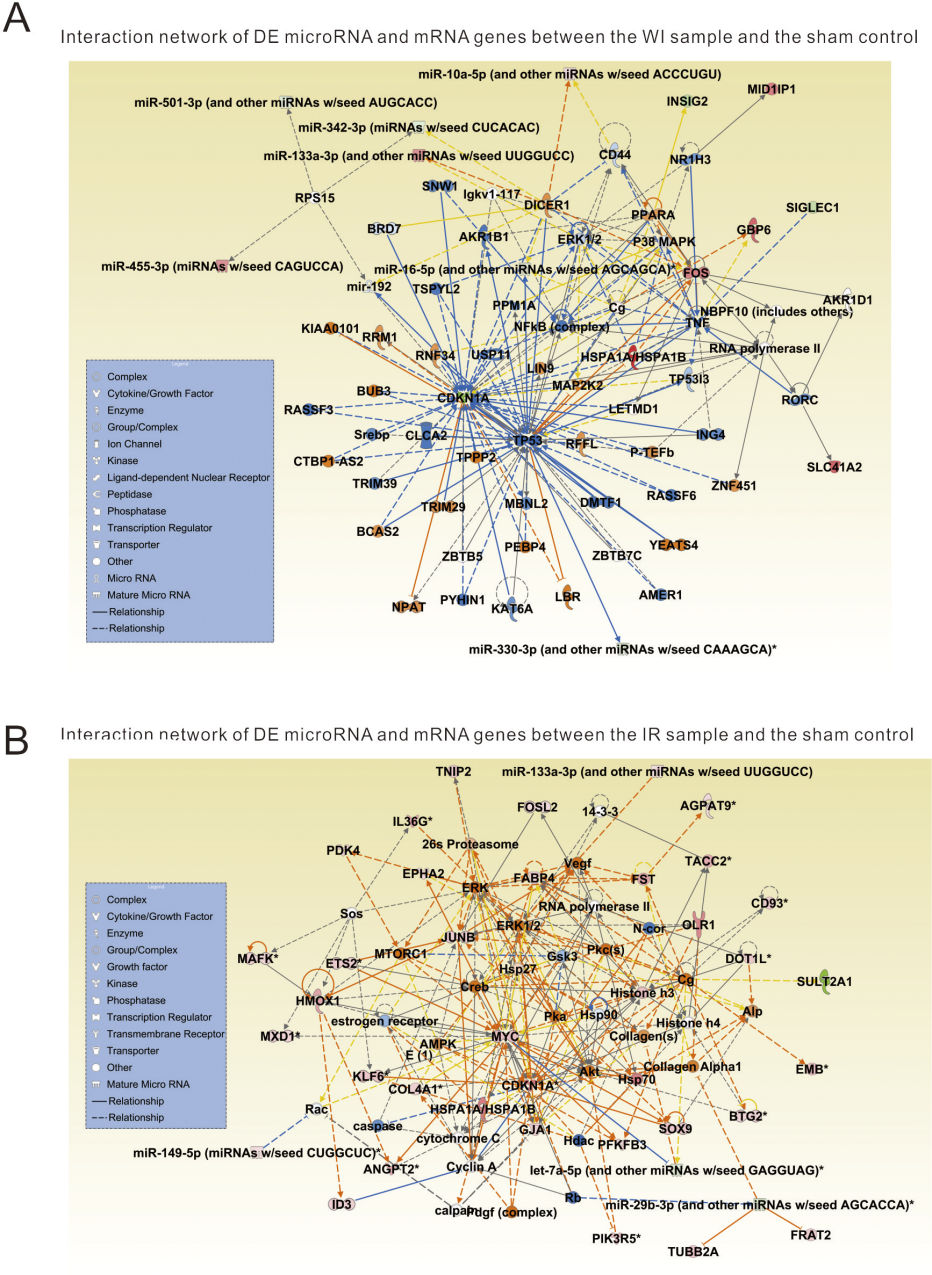
Interaction networks of the identified differentially expressed genes. (A) The network involves the DE microRNAs and mRNAs between the ischemia injury and the sham control. (B) The network involves the DE microRNAs and mRNAs between the ischemia followed reperfusion injury and the sham control. Genes are categorized as nodes with various shapes. Solid lines indicate direct interactions and dashed lines indicate indirect interactions.

In order to better understand the regulatory roles of microRNAs in mouse hepatic IR injury, we used the microRNA target filter function of Ingenuity Pathway Analysis (IPA, http://http://www.ingenuity.com/) to integrate the DE miRNAs and DE mRNA genes identified in the above analysis, then to identify their most relevant molecular networks and biological functions. The results show that the DE miRNAs and DE mRNA genes from the comparison of the WI sample and the sham sample are enriched in a network associated with decreased depolarization of mitochondria and mitochondrial function, liver proliferation and oxidative stress, etc. (Fig 4A). For example, a DE microRNA miR-195a-5p that was up-regulated in the WI sample compared to the sham sample, is associated with transmembrane potential of mitochondria and mitochondrial membrane [24], indicating ischemia likely starts to impair the function of mitochondria in liver cells. This suggests that the metabolism process may have been impaired at the ischemia stage and the proinflammatory molecules may be released. In contrast, the DE miRNAs and DE mRNA genes from the comparison of the IR sample and the WI sample are enriched in a network associated with activation of immune and inflammatory response pathways such as acute phase response signaling, IL-6 signaling, Toll-like receptor signaling and NF-κB signaling, etc. (Fig 4B). This implies that the acute and severe inflammatory responses occur at the reperfusion stage not at ischemia stage.

### Modes of microRNA expression change in hepatic livers in the treatment of ischemia followed by reperfusion

**Fig 5:**
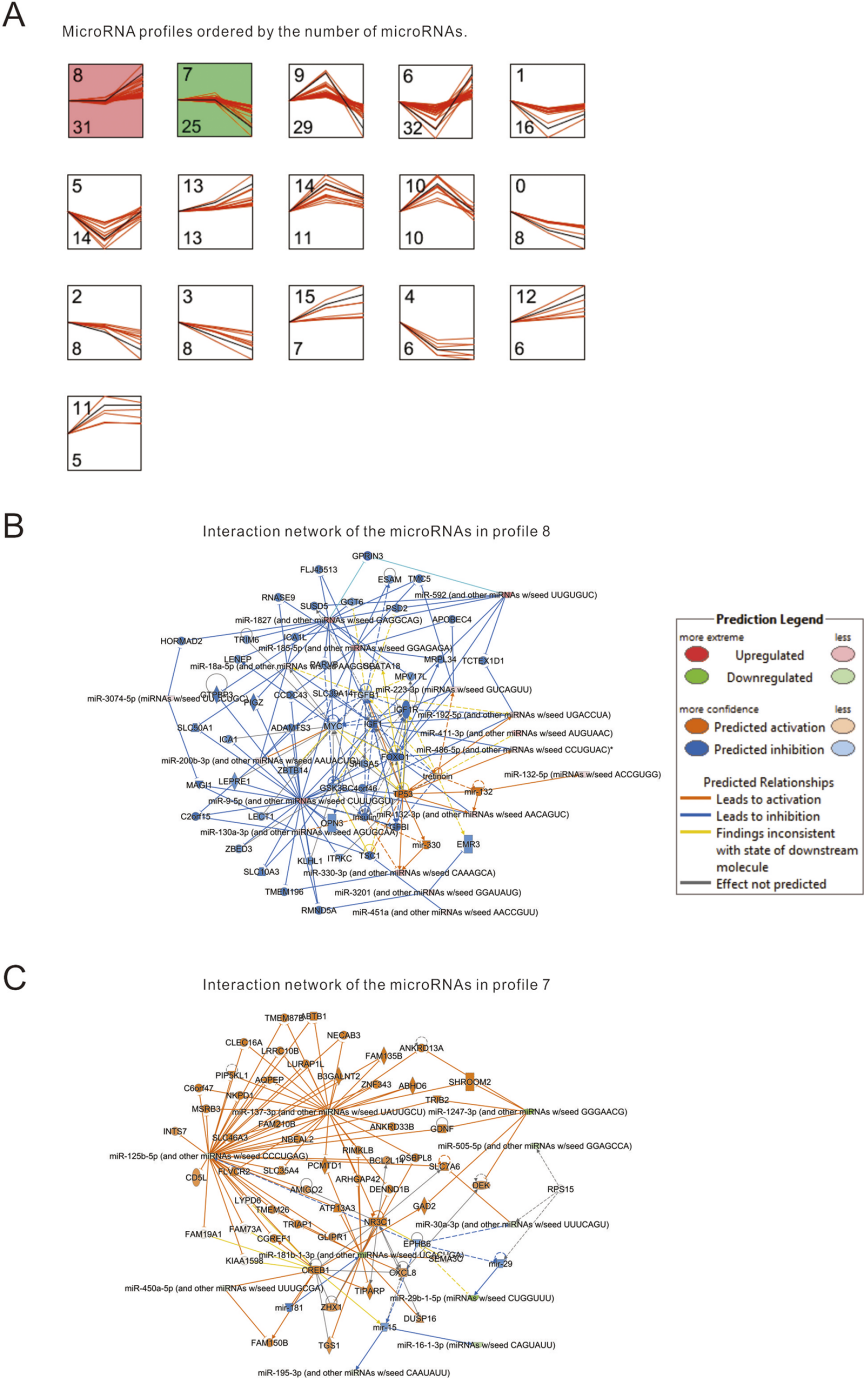
The modes of microRNA expression change in the hepatic IR injury. (A) Two significant profiles (profile 8 and profile 7) out of 16 profiles were detected as significant using STEM analysis. Redlines represent expression dynamics of individual microRNAs from sham, WI, and IR (left to right). Black curve represents the model of each microRNA expression profile. The numbers in the upper and lower left corners are the profile identifier and the miRNA count assigned to that profile. (B-C) The regulatory networks consist of the microRNAs in profile 8 and 7 and their target genes, respectively.

The above analysis showed that the WI injury and the IR injury were different in histological and transcriptomic perspective. In order to reveal the patterns of gene expression change, we attempted to identify different sets of genes consisting of distinct gene expression change mode by applying the tool STEM that was a specifically designed tool for the analysis of short time series gene expression data [25]. STEM analysis of microRNA profiles of the sham sample, the WI sample, and the IR sample identified 16 profiles (Fig 5A). There are two significant profiles (profile 8 and 7). Interestingly, both the two profiles displayed a similar expression level between the sham sample and the WI sample whereas the expression level was precipitously changed in the IR sample. These modes are consistent with the aforementioned findings that there are severe histological changes (Fig 1) and extensive disturbance in gene expression in the IR sample (Fig 3). This further suggests that reperfusion causes primary expression change and hepatic injury during the ischemia-reperfusion treatment.

We next used IPA tool to infer the biological functions of the microRNAs in the profile 7 and 8. The results showed that the microRNAs in the profile 8 are enriched in a biological network associated with cancer, organismal injury and abnormalities, etc. (Fig 5B). For example, miR-223 in the profile 8 was up-regulated in the hepatic IR injury. Consistently, a previous study showed an increased expression level of miR-223 in mouse liver with IR injury [18]. The microRNAs in the profile 7 are enriched in a biological network associated with connective tissue disorders, inflammatory disease and inflammatory responses (Fig 5C). Collectively, the results indicate that the perturbation in microRNA expression at reperfusion stage mainly contribute to inflammatory responses and disease. This is consistent with the previous findings [26, 27].

## Discussion

Hepatic IR injury is a complex physiological process involving intricate regulation of gene expression and protein interaction [6–8, 28]. MicroRNAs are short non-coding RNAs participating in regulation of many biological processes. Our study profiled expression of microRNA and mRNA genes in mouse livers at the three stages of IR injury (sham, ischemia, and reperfusion) and pairwisely compared the expression profiles. The results showed that reperfusion injury caused much more extensive perturbation in gene expression compared to ischemia injury and sham control. In consistence with this, there is more severe injury at the reperfusion stage of IR injury process. DE microRNAs at ischemia stage play a regulatory role in metabolism process and mitochondrial functions. In contrast, DE microRNAs at reperfusion stage mainly function in immune and inflammatory responses.

**Fig 6:**
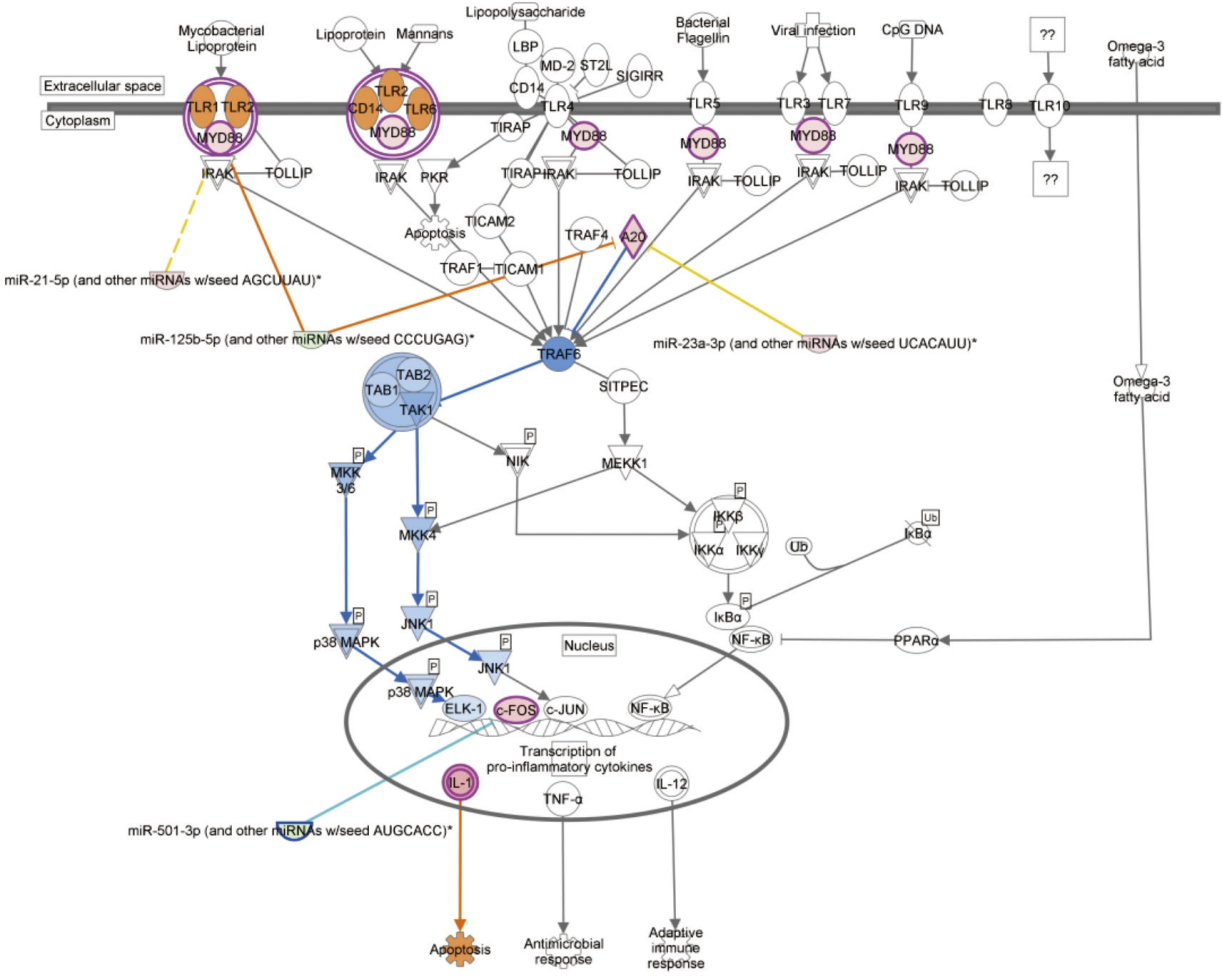
Toll-like receptor pathway in response to the hepatic IR injury. Two microRNAs miR-125p and miR-501-3p are down-regulated in IR injury when compared to the sham control. Consequently, their target genes *Myd88*, *A20*, and *c-Fos* are up-regulated and activate the Toll-like receptor pathway.

Our study separated ischemia stage from ischemia followed reperfusion for the first time, and profiled their gene expression, respectively. This design allowed us to investigate different injury to livers and molecular mechanisms of ischemia injury and reperfusion injury following ischemia. Indeed, our results show that 42% of the DE microRNAs between the reperfusion injury and the ischemia injury are not in the DE microRNAs between the IR sample and the sham sample. Moreover, ischemia and reperfusion injury affect expression of the different genes with distinct functional themes. Interestingly, when we directly compared gene expression profiles of the IR sample and the sham sample, we found that the DE genes (microRNAs and mRNAs) were associated with the Toll-like receptor signaling pathway in which miR-125b-5p and miR-501-3p were down-regulated at reperfusion stage, leading to the increased expression of their target *Myd88*, *c-Fos* and *A20* (Fig 6). The realtime quantitative PCR results confirmed the expression changes of the selected genes (S2 Fig). Consequently, it initiated immune and inflammatory responses to IR injury. A previous study also reported that Toll-like receptor signaling pathway was activated in hepatic IR injury [29]. However, previous studies didn’t separated ischemia stage, only obtained the ending effect of IR injury, and failed to provide a complete picture of ischemia / reperfusion injury.

## Supporting Information

S1 Fig Realtime quantitative PCR of selected differentially expressed microRNAs (A) and mRNAs (B). *** indicates p-value < 0.001, * indicates p-value < 0.05, t-test.
(JPG)

S2 Fig Realtime quantitative PCR of mmu-miR-501-3p and Fos involved in Toll-like receptor pathway. Both genes are differentially expressed between the IR sample and the sham sample (*** indicates p-value < 0.001, t-test). The expression changes of mmu-miR-501-3p and Fos are negatively correlated.
(JPG)

S1 Table The primers for qPCR of selected differentially expressed mRNAs. (DOC)

S2 Table The primers for qPCR of selected differentially expressed microRNAs. (DOC)

S3 Table The list differentially expressed microRNAs between the sham, WI, and IR samples. (XLS)

S4 Table The list differentially expressed mRNAs between the sham, WI, and IR samples. (XLS)
