# Supplementary figures and images for: Global MicroRNA Expression Profiling of Mouse Livers following Ischemia-Reperfusion Injury at Different Stages
Source: PLoS One. 2016 Feb 9;11(2):e0148677. doi: 10.1371/journal.pone.0148677 (PMC4747576; doi:10.1371/journal.pone.0148677)

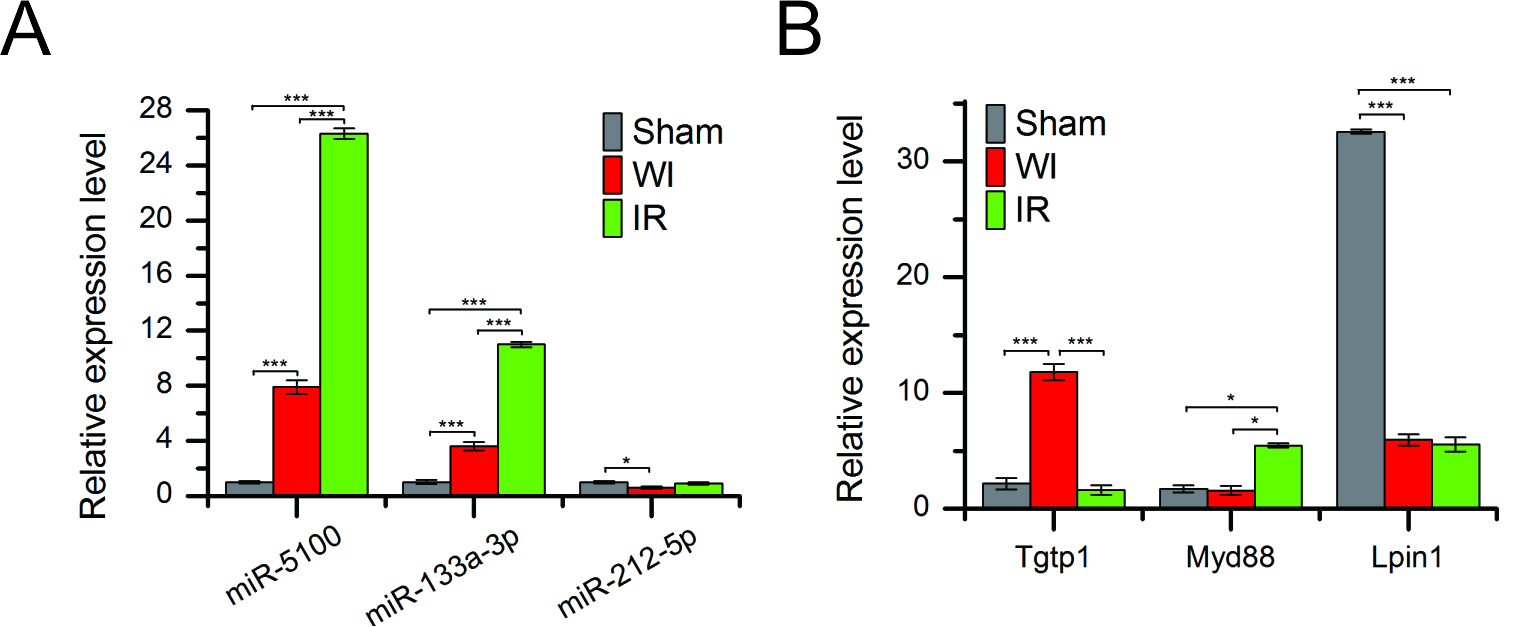

Supplement: S1 Fig — Realtime quantitative PCR of selected differentially expressed microRNAs (A) and mRNAs (B). *** indicates p-value < 0.001, * indicates p-value < 0.05, t-test. (JPG) [file pone.0148677.s001.jpg]

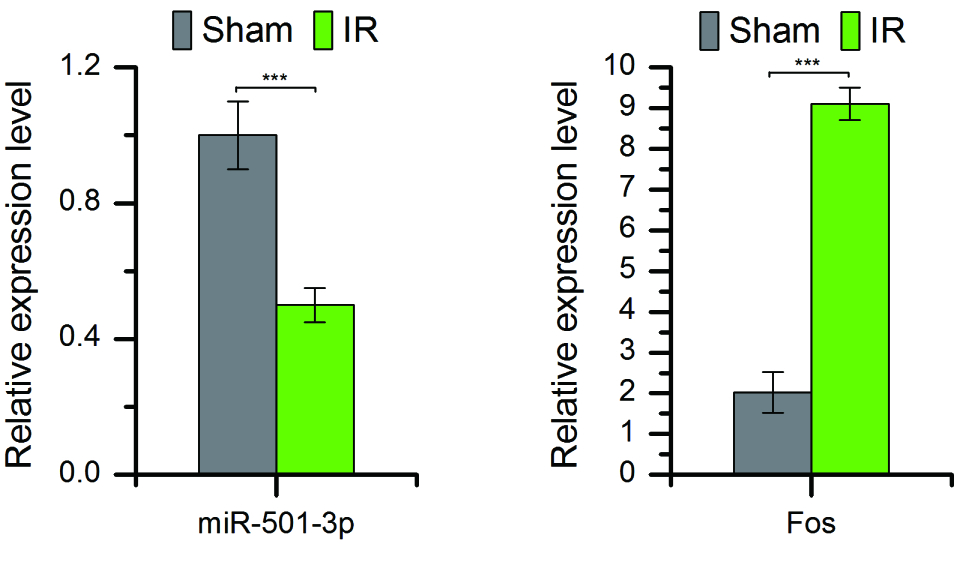

Supplement: S2 Fig — Both genes are differentially expressed between the IR sample and the sham sample (*** indicates p-value < 0.001, t-test). The expression changes of mmu-miR-501-3p and Fos are negatively correlated. (JPG) [file pone.0148677.s002.jpg]
