# Supplementary material for: Global MicroRNA Expression Profiling of Mouse Livers following Ischemia-Reperfusion Injury at Different Stages
Source: PLoS One. 2016 Feb 9;11(2):e0148677. doi: 10.1371/journal.pone.0148677 (PMC4747576; doi:10.1371/journal.pone.0148677)
Supplement: S1 Table — (DOC) [file pone.0148677.s003.doc]

**S1** Table. The primers for qPCR of selected differentially expressed mRNAs

| **RNA Name** | **Sequence(5'-3')** |
| --- | --- |
| Tgtp1-F | GGCTGGAGCATTAGCCACC |
| Tgtp1-R | TGTTCTGCAAACAAATGGGGAA |
| Myd88-F | GACCGTGAGGATATACTGAAGGA |
| Myd88-R | GGCCACCTGTAAAGGCTTCTC |
| Lpin1-F | GGCCCTCAACACCAAAAAGTG |
| Lpin1-R | CGCTGTGAATGGCCTGAAAAT |
| Fos-F | CGGGTTTCAACGCCGACTA |
| Fos-R | TGGCACTAGAGACGGACAGAT |
| GAPDH-F | TGGCCTTCCGTGTTCCTAC |
| GAPDH -R | GAGTTGCTGTTGAAGTCGCA |

GAPDH is used as the endogenous control.
