# Supplementary material for: Global MicroRNA Expression Profiling of Mouse Livers following Ischemia-Reperfusion Injury at Different Stages
Source: PLoS One. 2016 Feb 9;11(2):e0148677. doi: 10.1371/journal.pone.0148677 (PMC4747576; doi:10.1371/journal.pone.0148677)
Supplement: S2 Table — (DOC) [file pone.0148677.s004.doc]

**S2** Table. The primers for qPCR of selected differentially expressed microRNAs

| **RNA Name** | **Sequence(5'-3')** |
| --- | --- |
| mmu-miR-5100 | TCGAATCCCAGCGGTGCCTCT |
| mmu-miR-133a-3p | TTTGGTCCCCTTCAACCAGCTG |
| mmu-miR-212-5p | ACCTTGGCTCTAGACTGCTTACT |
| mmu-miR-501-3p | AATGCACCCGGGCAAGGATTTG |
| RnU6-F | CTCGCTTCGGCAGCACA |
| RnU6-R | AACGCTTCACGAATTTGCGT |

The common reverse primer for microRNA is ACGCCTGGAATGTAAAGAAGTATG. RnU6 is used as the endogenous control.
